# Dermatologic Manifestations of Mitochondrial Dysfunction: A Review of the Literature

**DOI:** 10.3390/ijms25063303

**Published:** 2024-03-14

**Authors:** Nicole Natarelli, Nimrit Gahoonia, Shaliz Aflatooni, Sahibjot Bhatia, Raja K. Sivamani

**Affiliations:** 1 Morsani College of Medicine, University of South Florida, 560 Channelside Drive, Tampa, FL 33602, USA; natarellin@usf.edu (N.N.); aflatooni@usf.edu (S.A.); 2 College of Osteopathic Medicine, Touro University, 1310 Club Dr, Vallejo, CA 94592, USA; ngahooni@student.touro.edu; 3 College of Medicine, California Northstate University, 9700 W Taron Dr, Elk Grove, CA 95757, USA; ssbhatia99@gmail.com; 4 Integrative Skin Science and Research, 1495 River Park Drive, Sacramento, CA 95819, USA; 5 Pacific Skin Institute, 1495 River Park Dr Suite 200, Sacramento, CA 95815, USA; 6 Department of Dermatology, University of California-Davis, 3301 C St #1400, Sacramento, CA 95816, USA

**Keywords:** mitochondria, dermatology, UVR, aging, hair loss, wound healing, lupus, psoriasis, vitiligo, atopic dermatitis

## Abstract

Mitochondria are eukaryotic cellular organelles that function in energy metabolism, ROS production, and programmed cell death. Cutaneous epithelial and hair follicle dermal papilla cells are energy-rich cells that thereby may be affected by mitochondrial dysfunction and DNA mutation accumulation. In this review, we aimed to summarize the medical literature assessing dermatologic conditions and outcomes associated with mitochondrial dysfunction. A search of PubMed and Embase was performed with subsequent handsearching to retrieve additional relevant articles. Mitochondrial DNA (*mtDNA*) deletions, mutation accumulation, and damage are associated with phenotypic signs of cutaneous aging, hair loss, and impaired wound healing. In addition, several dermatologic conditions are associated with aberrant mitochondrial activity, such as systemic lupus erythematosus, psoriasis, vitiligo, and atopic dermatitis. Mouse model studies have better established causality between mitochondrial damage and dermatologic outcomes, with some depicting reversibility upon restoration of mitochondrial function. Mitochondrial function mediates a variety of dermatologic conditions, and mitochondrial components may be a promising target for therapeutic strategies.

## 1. Introduction 

### 1.1. Mitochondria Overview 

Mitochondria are maternally inherited eukaryotic cellular organelles with an important role in energy metabolism. They are composed of an enzyme-filled matrix surrounded by two membranes and an intermembrane space [1]. Distinct metabolic processes occur in various mitochondrial compartments, and proper compartmentalization is necessary for function.

Mitochondria-residing metabolic processes include the citric acid cycle and oxidative phosphorylation. The citric acid cycle is a series of chemical reactions in the mitochondrial matrix that functions to oxidize carbohydrate, fat, and protein-derived acetyl-CoA. In the process, coenzymes NAD+ and FAD are reduced for further electron transport [2], and guanosine triphosphate (GTP) molecules are produced via substrate-level phosphorylation. These GTP molecules can then be converted to adenosine triphosphate (ATP) molecules, the primary energy source for a variety of biochemical and physiologic processes [3].

The reduced coenzymes NADH and FADH_2_ can then enter the electron transport chain and contribute to ATP production via oxidative phosphorylation. Oxidative phosphorylation is the most efficient ATP-producing metabolic pathway, characterized by movement of electrons through the mitochondrial electron transport chain, pumping of protons against the concentration gradient, and utilization of the resulting electrochemical gradient to fuel ATP production [4].

Besides having a large role in energy production for cells, mitochondria are distinctly unique from other organelles as they carry their own mitochondrial DNA (*mtDNA*). Damage to *mtDNA* has been shown to mediate the disease process of various conditions such as aging, cancer, and mitochondria-specific disorders. *mtDNA* damage may be secondary to genetic mutations in *mtDNA* that disrupt genes encoding mitochondrial proteins involved in energy production. Furthermore, environmental exposure to UV radiation may induce oxidative stress, which can damage both nuclear and *mtDNA* via ROS overwhelming antioxidant defense [5]. Unlike nuclear DNA, *mtDNA* lacks association with protective histone proteins and lacks efficient nuclear repair mechanism [6]. Consequently, mitochondria are vulnerable to damage inflicted by various stressors, with an increased mutation rate compared to nuclear DNA [7]. A lack of recent data necessitates further investigation into the mechanism of mitochondrial dysfunction and key signaling pathways mediating skin disease.

However, in addition to retrograde signaling, mitochondria have multiple endogenous DNA repair mechanisms, including base excision repair (BER), the most widely understood repair mechanism. Mitochondrial base excision repair tends to be highly efficient as many of the required proteins are located in the nuclear and mitochondrial compartments, with a specific subset being highly concentrated in the mitochondria [8]. The involved proteins include glycosylases (recognize and remove the damaged base), APE1 (incision), POLG and POLB (gap filling), and LIGIII (ligation) [9]. Proteins such as transcription and packaging factor (TFAM) and PARP1 ones prevent base excision repair activation. Another pathway, the mismatch repair pathway (MMR), was previously poorly studied as a repair mechanism in mitochondria. However, recent data have supported the presence of a mitochondrial MMR that is distinct from that of the nuclear pathway [10]. The YB-1 factor is believed to play a significant role in mitochondrial MMR and is understood to function to recognize and bind to mismatch repair [10]. Recent evidence also supports the presence of double-stranded break repair in mitochondrial cells through homologous recombination and nonhomologous end joining. Finally, it is currently believed that there is no presence of nucleotide excision repair in mammalian mitochondria [10].

In addition to DNA repair mechanisms, mitochondria also display mitophagy. Mitophagy is a self-degradation process that occurs when mitochondria become dysfunctional as noted by the accumulation of reactive oxygen species, *mtDNA* mutation accumulation, abnormal mitochondrial morphology, and decreased ATP-producing capacity [11]. The process of initiating and implementing mitophagy is extremely complex, involving several pathways. At a basic level, it is thought that upon the expression of certain cues by dysfunctional mitochondria, mitophagy receptors are activated to be expressed on the mitochondrial surface. This leads the path for autophagosomes to target and destroy the unhealthy mitochondria with the help of lysosome fusion [12]. The main mitophagy receptors that are outlined in mammalian mitochondria include BNIP3, BNIP3L, and FUNDC1 [9].

#### Role of Mitochondria in Skin

The skin is characterized by a high rate of turnover, requiring ample energy. Energy is supplied by mitochondrial processes, primarily within the electron transport chain [13]. This supports various functions such as cell signaling, wound healing, and hair growth [14]. Mitochondria also play a vital role in allowing the skin to effectively maintain microbial defense function and epidermal homeostasis [14]. Furthermore, proliferation of the skin, characterized by keratinocyte differentiation from basal to cornified keratinocytes followed by migration to the outer layer of skin, requires input from mitochondria via downstream transcriptional factors including C/EBP, Notch, and beta-catenin which together increase the differentiation of murine embryonic stem cells and induced pluripotent stem cells, as well as tissue-specific multipotent epithelial stem cells [13]. A study described by a recent review article highlighted the importance of mitochondrial transcription factor A in primary keratinocyte differentiation in a mouse model study; the mice without the transcription factor furthermore had low ROS levels, suggesting a delicate balance between mitochondrial respiration and ROS production for the process of skin proliferation [13]. Keratinocyte proliferation is additionally dependent on Ca^2+^ uptake by the mitochondria, with in vitro studies showing that cells with higher Ca^2+^ levels had better differentiation capacity [13].

Unhealthy mitochondria are involved in chronological skin aging according to the literature. The *mtDNA* genome, specifically a region of 4977 base pairs encoding electron transport chain complexes, has been shown to be commonly deleted in older human skin [13]. Studies have also shown that this region in the genome is more frequently deleted in those over 70 years of age and in subjects with higher sun exposure [13]. Mitochondria are key players in photo-aged skin as well. UV exposure from the sun causes damage to *mtDNA* as well as pathogenic oxidative stress to skin cells [13]. Many separate studies have found certain regions of *mtDNA* to be deleted in UV-exposed skin which downstream leads to increased activity of collagen-degrading enzymes, decreased expression of collagen synthesis genes, and oxidative stress; collectively, these factors cause the wrinkled appearance of photo-aged skin [13]. Furthermore, well-known is the role of UV damage in the development of skin cancer. Similar to photo-aged skin, skin cancer cells have also been shown to have significantly altered mitochondria [13]. Deletions in the *mtDNA*, excessive ROS production, and mitochondrial apoptosis have each been evident in studies conducted on skin cancer cells [13]. Therefore, while mitochondria are known to provide skin with the necessary energy required to function, any deviation in their functionality may contribute to manifestations of underlying disorders.

Considering the integral role of the mitochondria in the maintenance of skin, it is not surprising that many dermatological conditions have been shown to have disturbed mitochondrial structure and function. Systemic lupus erythematous (SLE), an autoimmune disease with significant skin involvement, has been shown in studies to have altered mitochondrial function. Specifically, excessive production of ROS by mitochondria and dysfunctional mitochondrial antioxidant enzymes present in the immune cells of SLE patients has been found to subject patients with SLE to inflammatory responses [15]. Psoriasis is also a condition with established evidence of mitochondrial dysfunction contributing to pathogenesis. Mitochondria in psoriasis have been shown to upregulate ROS production, increase downstream inflammatory cytokines, and increase dendritic cell activation [16,17]. Similarly, vitiligo, a condition involving dysfunctional melanocytes, has altered mitochondrial function. Common to this disease pathway as well is overproduction of ROS by mitochondria and abnormal ETC function characterized by proton leaks in vitiligo melanocytes [18]. Furthermore, defective mitochondrial size and cristae structure have been implicated in lesional vitiligo skin [19]. Studies have shown that mitochondrial disturbances are present in atopic dermatitis (AD). The high ROS levels and proton leaks, similar to vitiligo, have been noted in nonlesional AD keratinocytes as well [20]. Each of these dermatologic conditions presents compelling evidence of mitochondrial dysfunction playing a significant role in their pathogenesis and manifest in distinct manners that demand attention.

In this review paper, we aim to summarize the role of dysfunctional mitochondria in various dermatological conditions and discuss potential therapeutic strategies. Original research was conducted in PubMed and Embase using controlled vocabulary and keywords including “mitochondria”, “aging”, “hair loss”, “wound healing”, “lupus”, “psoriasis”, “vitiligo”, and “atopic dermatitis”. Relevant studies of any type, including murine and in vitro studies, were retrieved. Papers were not excluded based on publication date, although preference was given to more recent publications. The search and data abstraction were completed by four authors and were not approached systematically.

## 2. Mitochondria Dysfunction and Dermatological Manifestations

### 2.1. Mitochondria and Cutaneous Aging

The relationship between dysfunctional mitochondria and aging phenotypes has been widely established in a variety of tissues. For example, two mouse model studies observed signs of premature aging with elevated levels of mitochondrial DNA (*mtDNA*) mutations [21,22]. In 2005, authors observed accelerated aging in mice with a proofreading-deficient mitochondrial DNA polymerase (POLG) and resulting *mtDNA* mutation accumulation [21]. Interestingly, they did not observe increased markers of oxidative stress with *mtDNA* mutation accumulation. However, mutations correlated with increased levels of apoptotic markers. The levels of apoptotic markers also increased in normal aging mice, leading the authors to conclude that the promotion of apoptosis due to *mtDNA* mutation accumulation may promote mammalian aging [21].

Similarly, Trifunovic et al. observed a reduced lifespan and premature onset of aging phenotypes in mice with increased levels of point mutations and deleted *mtDNA* [22]. They similarly created knock-in mice expressing a proofreading-deficient mitochondrial DNA polymerase, resulting in a threefold to fivefold increase in *mtDNA* point mutations. Premature aging phenotypes included weight loss, reduced subcutaneous fat, alopecia, kyphosis, osteoporosis, anemia, reduced fertility, and heart enlargement [22]. These studies established a causative link between *mtDNA* mutation accumulation and the onset of premature aging. In addition, novel studies have assessed the role of mitochondrial dysfunction specifically in cutaneous aging.

Cutaneous aging is intrinsically characterized by the chronological loss of collagen, atrophy of the dermis, degeneration of the elastic network, and loss of hydration [23]. Significant epidermal thinning and loss of regenerative capacity occur with aging, the molecular basis of wrinkle formation stems from the fragmentation of collagen and the reduction of other functional components including elastin, glycosaminoglycans, and proteoglycans. This process is further impacted by extrinsic environmental factors, such as ultraviolet radiation, with photoaged skin characterized by deeper wrinkles and uneven pigmentation [13]. Several studies have demonstrated that cutaneous aging is associated with decreased mitochondrial function; conversely, mitochondrial dysfunction can promote premature cutaneous aging via wrinkle formation, uneven pigmentation, and uneven wound healing [14].

#### 2.1.1. Chronological Aging Is Associated with Decreased Mitochondrial Function

Chronological skin aging is characterized by decreased mitochondrial function, exemplified via loss of membrane potential, nuclear and mitochondrial gene mutations, and electron transport chain defects [13]. For example, a study observed an age-dependent increase in the incidence of a 4.977 bp *mtDNA* deletion, known as the “common deletion” in normal skin [24]. In addition, altered mitochondrial physiology is associated with elevated reactive oxygen species (ROS), which is further associated with factors that may accelerate aging processes, such as changes in protein structure and function, gene expression, and DNA [25]. Researchers propose that increased mitochondrial dysfunction may be due to age-dependent mitochondrial damage and an age-dependent reduction in dysfunctional mitochondria removal, termed mitophagy [26]. However, recent evidence suggests that clonal expansion of *mtDNA* replication errors may account for the accumulation of aging-associated *mtDNA* mutations, rather than sole damage accumulation [27].

A 2014 study observed significant differences in the expression of 137 genes in human dermal fibroblasts of old female donors vs. young female donors, with variance in mitochondrial gene expressions being the most notable [28]. Similarly, a 2011 study analyzed mitochondrial function in dermal fibroblasts obtained from human donors stratified by age [29]. As proteasomes are involved in the degradation of oxidized proteins during aging, the authors used a degron-destabilized green fluorescent protein (GFP)-based reporter protein to assess proteasome activity. Older donors depicted a significant decrease in mitochondrial membrane potential, a significant increase in ROS levels, and variability in proteasome activity. Yet, fibroblasts from middle-aged donors depicted significantly reduced proteasome activity compared to young donors. Lastly, the authors observed interdependence of mitochondrial and proteasomal activity: inhibition of the proteasome resulted in decreased mitochondrial function and vice versa [29]. These results suggest that mitochondrial membrane potential, a key marker of mitochondrial activity, decreases as a function of cutaneous aging. Similarly, a 2008 study found the energy metabolism of aging skin to be functionally aerobic, signifying the functional loss of mitochondria in chronological cutaneous aging [25]. Furthermore, a study found keratinocytes in the old skin to exhibit a significantly more fragmented network than keratinocytes in the young skin [30]. Lastly, a 2023 review summarizing original research concluded that a declined oxidative phosphorylation state and decreased mitochondrial integrity were more often found in fibroblasts of older subjects than in those of younger subjects [31].

Complex II of the electron transport chain has been particularly implicated in the mitochondrial theory of aging [22]. A 2016 study analyzed complex II activity in cutaneous fibroblasts and keratinocytes from donors of various ages. An age-related decrease in fibroblast complex II activity was observed. Transcript expression and protein levels of both complex II catalytic subunits A and B were significantly decreased in older fibroblast samples [32]. In contrast, the authors observed no decrease in complex IV activity with increasing age, suggesting mitochondrial functional loss may be localized to specific complexes of the electron transport chain, including complex II.

In addition to complex II, Coenzyme Q, an endogenously produced antioxidant, is an integral part of the electron transport chain; it accepts electrons from reducing equivalents and transfers them to electron acceptors, fostering biochemical ATP generation. An age-related impairment in Coenzyme Q_10_ has been observed in various organs, including the skin [33]. Furthermore, an in vitro study demonstrated CoQ_10_ inhibition increases oxidative stress and mitochondrial dysfunction and contributes to premature aging in human dermal fibroblasts [34].

#### 2.1.2. Mitochondrial Dysfunction and Cutaneous Aging

Interestingly, a variety of genetic disorders of mitochondrial dysfunction are characterized by premature aging [35]. For example, Cockayne Syndrome, a condition characterized by nucleotide excision repair deficits, mitochondrial dysfunction, and defective mitophagy, leads to a variety of dermatological manifestations associated with cutaneous aging such as photosensitivity, pigmentation, scarring, inflammation, anhidrosis, and epidermal thinning [35]. Similarly, mitochondrial dysfunction, reduced mitophagy, and increased mitochondrial ROS are components of Werner Syndrome, which is associated with skin wrinkling and abnormal pigmentation [35]. Although these observations depict an association between mitochondrial dysfunction and premature aging, few studies have established causality.

However, a 2018 mouse model study evaluated the consequences of *mtDNA* depletion in various tissues, including the skin [36]. *mtDNA*-depleted mice depicted decreased mitochondrial gene expression and instability of oxidative phosphorylation complexes, resulting in reduced oxidative phosphorylation enzymatic activities. The authors observed visible skin wrinkling and hair loss in *mtDNA*-depleted mice. Skin wrinkling was further associated with epidermal hyperplasia, hyperkeratosis, increased matrix metalloproteinase expression, and decreased matrix metalloproteinase inhibitor expression. Furthermore, the authors demonstrated that downregulating mutant transgene expression restored mitochondrial function, as well as skin and hair integrity [36]. These results suggest that (1) mitochondrial function is intrinsically linked to factors implicated in skin aging, such as skin inflammation and wrinkling, and (2) restoration of mitochondrial function may reverse skin pathology. Although this study is conducted in a mouse model, it demonstrates the importance of proper mitochondrial function in cutaneous homeostasis and aging prevention.

#### 2.1.3. Mitochondrial Dysfunction and Photoaging

Ultraviolet (UV) radiation is a significant cause of premature skin aging due to DNA and stem cell damage in addition to increased oxidative stress [37]. UV radiation can promote ROS production via a multitude of mechanisms, such as by affecting catalase enzymes, up-regulating nitric oxide synthase synthesis, decreasing protein kinase C expression, and DNA modification [38]. As a large intracellular source of oxidants, mitochondria are particularly susceptible to ROS and DNA damage [39], making mitochondria an important target of UV radiation.

For example, sun-exposed areas depict a greater accumulation of *mtDNA* deletions than protected areas of skin [14]. Whereas a previously described study observed an age-dependent increase in the “common deletion” in normal skin, an intraindividual comparison study also observed a 10-fold increase in the common deletion in photoaged skin compared to sun-protected skin [40]. In addition, the incidence of UVA-induced *mtDNA* deletions increases in the skin affected by earlier UV radiation, regardless of whether additional UVA exposure is sustained [41].

*mtDNA* deletions are associated with phenotypic features of photo-aged skin. A 2011 study observed that *mtDNA*-depleted skin fibroblasts depicted increased levels of collagen-degrading metalloproteases and a downregulation of collagen biosynthesis genes [42], which is a clinically significant finding as decreased collagen promotes wrinkle formation. Thus, in addition to serving as a biomarker for photoaged skin, *mtDNA* deletions may directly contribute to the biochemical processes underlying cutaneous photoaging.

### 2.2. Mitochondrial Dysfunction and Wound Healing

As an energy-rich process, proper wound healing necessitates adequate mitochondrial function. A 2021 study observed amplified mitochondrial activity in injured swine tendons compared to control tendons following a rotator cuff injury [43]. Injured tendons depicted higher protein expression of citrate synthase and complex I, in addition to increased mitochondrial density. Furthermore, studies have assessed mitochondrial function in cutaneous wound healing. A 2021 mice model study analyzed mitochondrial metabolism in early- and late-stage skin macrophages and observed stage-specific changes in mitochondrial metabolism [44]. Whereas early-stage wound macrophages were characterized by mitochondrial ROS production and HIF1α stabilization, promoting a pro-angiogenic process necessary for proper wound healing, late-phase resolving macrophages were marked by IL-4Rα-mediated mitochondrial respiration [44]. ROS production of early-stage wound macrophages mediates HIF1α-induced VEGF-A expression and thus generates an appropriate vascular response necessary for timely healing. Collectively, these studies highlight the integral role of proper mitochondria function in various stages of both tendon and cutaneous wound healing.

Mitochondrial dysfunction has been associated with delayed wound healing in diabetic mice [45]. As diabetes can delay wound healing, researchers analyzed mitochondrial metabolic indices via optical fluorescence imaging and the volumetric redox state of wound tissue via 3D optical cryo-imaging. Fluorescence cryo-imaging provides a 3D metabolic state of tissue, allowing for users to obtain redox information. Freezing techniques provide adequate immobilization of the sample in a pseudo-native state for imaging. Diabetic wounds have been found to exhibit greater mitochondrial dysfunction and greater oxidative stress compared to wounds in control mice [45]. This is an anticipated finding, as oxidative stress has been widely implicated in the pathogenesis of delayed wound healing among diabetics [46].

However, the role of mitochondria dysfunction in delayed wound healing has yet to be fully understood. A mice model study observed age-dependent effects of a Sod2-deletion mouse model that generates mitochondrial oxidative stress in epidermal stem cells, with resulting cellular senescence and arrested keratinocyte proliferation [47]. Interestingly, Sod2 deficiency *accelerated* wound closure in young mice with increased epidermal differentiation and reepithelialization. However, old mice depicted delayed wound closure and reduced epidermal thickness with Sod2 deficiencies. These results suggest that the mitochondrial function may mediate skin quality and wound closure in an age-dependent manner. The authors further suggest such age-dependent effects of mitochondrial dysfunction may contribute to the age-dependent decline in epidermal regeneration [47].

#### Potential Therapeutic Strategies

Although the precise mechanism of mitochondrial-mediated wound healing has yet to be fully elucidated, a variety of studies have targeted mitochondrial dysfunction as a potential therapeutic strategy in impaired wound healing. Specifically, strategies promoting low oxidative stress and ROS production have shown promising results, as low concentrations of ROS are necessary for protection against invading microorganisms. However, excessive ROS production contributes to oxidative damage and delayed wound healing [48]. A mouse model study found that a mitochondria-targeted antioxidant improved dermal wound healing and closure and stimulated epithelization, granulation tissue formation, and vascularization [46]. Furthermore, as elamipretide, a mitochondrial-targeted antioxidant that reduces ROS production and stabilizes mitochondrial function, has been shown in human studies to improve heart failure and mitochondrial myopathy, researchers speculate that elamipretide may similarly improve chronic wound healing [48]. Additional studies are ultimately required to test this hypothesis, although preliminary research suggests a beneficial role of mitochondria-targeted antioxidants in the mitigation of mitochondrial dysfunction and aberrant inflammatory responses in impaired wound healing.

Interestingly, a 2019 study found that photobiomodulation with an 808 nm diode laser light led to an increased cell proliferation rate and migration ability. Increased ROS production was observed without a significant increase in oxidative stress-activated processes, such as lipid peroxidation and increased mitochondrial oxygen consumption and ATP synthesis [49]. The authors suggest the wound healing rate was increased due to ROS-mediated stimulation of mitochondrial activity. They found that ROS production was seen to be at its highest level 30 min after irradiation and decreased thereafter, which allowed for a shift from anaerobic to aerobic metabolism. This shift is necessary to promote cell proliferation. The authors hypothesized that this short-term increase still promotes low, rather than high, ROS concentration as low concentrations play a beneficial effect on signaling cascades. This study described a potential new mechanism in the ability of the 808 nm diode laser to protect against endothelial dysfunction while highlighting the utility of mitochondria modulation in the stimulation of effective wound healing.

Lastly, a 2023 review article detailed the correlation of burn injury progression with mitochondrial homeostasis, which is mediated via burn injury-induced hyperinflammation and subsequent mitochondrial dysfunction and cell death [50]. As mitophagy maintains cellular homeostasis by the selective removal of damaged mitochondria, the authors suggested that the process of mitophagy could be a promising therapeutic strategy for wound healing and burn injury. Furthermore, the authors discuss Pink1 and Parkin activators and USP30 inhibitors as potential therapeutic agents for the regulation of mitophagy and the subsequent mitigation of burn injury progression [50].

### 2.3. Specific Dermatologic Disorders with Mitochondrial Involvement

#### 2.3.1. Systemic Lupus Erythematosus

Several dermatologic conditions have been linked with aberrant mitochondrial activity. Systemic lupus erythematosus (SLE) is an autoimmune disease that has multiorgan system involvement, with the skin being one of the most likely affected organs; however, its phenotype can vary from individual to individual. Some of the most common SLE-specific skin manifestations include malar or discord rash, photosensitivity, and palatal ulcers [51]. For instance, in systemic lupus erythematosus (SLE), abnormal T-cell proliferation and death are dependent on mitochondrial production of reactive oxygen intermediates (ROI) and ATP synthesis [52]. One study specifically found the T cells of those with SLE to contain mitochondria with excessive ROS production, low glutathione and ATP levels, and hyperpolarized transmembrane potentials leading to increased susceptibility of T-cell death [52].

A 2016 study analyzed the role of oxidized mitochondrial nucleoids in SLE [53]. Healthy human neutrophils help to guide oxidized *mtDNA* to lysosomes for degradation after dissociation from transcription factor A mitochondria (TFAM). In SLE, the dissociation of *mtDNA* from the TFAM does not occur, resulting in oxidized nucleoids accumulating. Eventually, the accumulated oxidized *mtDNA* is extruded, triggering a type I interferon response commonly found in SLE [53].

Antibodies against DNA are a common finding in the blood of patients with SLE. The source of the autoantigens has not been clearly understood. Melki et al. explored the capacity of platelets to extrude *mtDNA* through the stimulation of FcγRIIA [54]. In lupus-prone transgenic mice, the stimulation of FcγRIIA led to platelet activation and release of *mtDNA*, leading to acceleration and aggravation of the lupus phenotype. This finding suggests that mitochondrial release by platelets is a major source of autoantigens in patients with SLE [54].

A 2021 study found that the programmed mitochondrial removal, typical in erythropoiesis, is defective in SLE [55]. SLE patients have an accumulation of red blood cells carrying mitochondria due to defective HIF-2α degradation. Macrophages engulf the mitochondria carrying red blood cells, resulting in *mtDNA* activating cycle GMP-AMP synthase (cGAS) in macrophages and inducing type I interferon production [55].

Lastly, a 2020 review summarizing mitochondrial dysfunction and SLE found lupus low-density granulocytes had increased mitochondrial ROS, promoting *mtDNA* oxidation and neutrophils extracellular trap (NET) formation [56]. NETs were found to increase interferogenic responses in target cells via the cyclic guanosine monophosphate–adenosine monophosphate synthase (cGAS) Stimulator of Interferon Genes (STING) pathway, contributing to lupus-like disease. Furthermore, the article discussed aberrant oligomerization of voltage-gated anion channels in the mitochondria outer membrane during oxidative stress, which causes short *mtDNA* fragments and the release of the DNA into the cytoplasm [56]. This further promotes the activation of the cGAS-STING path and upregulates type 1 interferon-stimulated genes. Interestingly, human SLE mononuclear cells have been found to contain increased levels of these voltage-gated anion channels. Ultimately, this suggests that pathological oxidative stress can contribute to immunological imbalance and to SLE pathogenesis.

#### 2.3.2. Psoriasis

Keratinocyte proliferation and chronic inflammation are components of the psoriasis diseases process, causing the presence of thick red or silvery patches on the skin. However, the reason behind this occurrence is not well understood [57]. Mitochondrial involvement has also been suggested in the pathophysiology of psoriasis. In one study, punch biopsies from healthy skin and psoriatic skin lesions were analyzed for the gene expression of uncoupling protein 2, dynamin-related protein 1, and calcineurin which are all regulators of mitochondrial function; the gene expression of each was found to be significantly decreased in patients with psoriasis compared to controls [57]. Additionally, the collected serum samples displayed significantly increased *mtDNA* in those with psoriasis [57]. Overall, the study suggested these pathological findings could be attributed to the inflammation and keratinocyte proliferation involved in psoriasis.

Mizuguchi et al. studied the role of mitochondrial ROS in the development of psoriatic inflammation [16]. A mouse model of imiquimod (IMQ)-induced psoriasiform skin inflammation was used to conclude that genetic deletion of p32/C1qpb, a regulator of mitochondrial protein synthesis and metabolism, protected the mice from IMQ-induced psoriatic inflammation. IMQ stimulation resulted in the production of mitochondrial ROS and promoted the production of inflammatory cytokines, as well as dendritic cell maturation. Dendritic cell activation is known to exacerbate psoriasis. The inhibition of mitochondria ROS using mitoquinone (MitoQ) decreased the severity of psoriasis in the mouse model through reduction in mitochondrial ROS and suppression of IMQ-induced dendritic cell activation. The authors concluded that p32/C1qbp may be an important genetic factor involved in psoriasis and mitochondrial ROS inhibition [16].

A 2021 study investigated the role of GDAP1L1 signaling implicated in mitochondrial fission in macrophages and the development of psoriasiform inflammation [17]. THP-1 cells stimulated by IMQ were utilized as the activated macrophage model. IMQ was also used to produce psoriasis-like lesions in a mouse model. After IMQ stimulation, increased expression of pro-inflammatory mediators and GDAP1L1 was observed in a transcriptomic assay of macrophages. GDAP1L1 regulated cytokine production through activating the phosphorylation of mitogen-activated protein kinases (MAPKs) and nuclear factor (NF)-κB pathways. Further, the silencing of GDAP1L1 inhibited cytokine release by macrophages and reduced the psoriasiform severity score from eight to two, suggesting macrophages play an important role for development and maintenance of psoriasiform lesions. The study demonstrated that mitochondrial fission factor GDAP1L1 can be targeted to reduce psoriatic inflammation [17].

A 2022 study also found that the *mtDNA* copy number is a potential biomarker for psoriasis [58]. In the case–control study, *mtDNA* copy number was significantly decreased in patients with psoriasis compared to healthy controls. Sub-group analysis revealed that *mtDNA* copy number was significantly lower in patients with a longer psoriasis diagnosis and significantly lower in patients with higher disease severity. The study suggests that *mtDNA* copy number can be a non-invasive biomarker for early detection of psoriasis [58].

However, it is necessary to note that increased *mtDNA* copy number can be indicative of mitochondrial dysfunction resulting from oxidative stress, aging, genetic mutations, or exposure to toxins. Such stressors result in increased mitochondrial biogenesis to prevent genomic instability [59]. A recent study investigating the diagnostic role of *mtDNA* copy number in alopecia areata patients found significantly elevated *mtDNA* copy number among cases, highlighting its potential use in diagnosing and evaluating alopecia severity [60]. In addition, another study found an increased *mtDNA* copy number in patients with vitiligo compared to healthy controls [61]. Thus, although authors observed decreased *mtDNA* copy number among psoriasis patients, this observation must be interpreted cautiously, and future research is necessary to evaluate the association of copy number and psoriasis incidence and severity.

#### 2.3.3. Vitiligo

Another common dermatologic condition, vitiligo, has also been shown to have mitochondrial influence. Vitiligo is a skin condition in which there is a loss of function of melanocytes causing white skin lesions. The cause of this loss of function is not well understood [18]. A study in 2022 found abnormalities in mitochondrial ATP production with compensatory increases in glycolytic enzymes (hexokinase II, pyruvic dehydrogenase kinase 1, and pyruvic kinase M2) in cultured epidermal vitiligo melanocytes compared to healthy melanocytes [18]. The authors also found vitiligo melanocytes to have the highest proton leak, suggesting an impaired ETC function, and despite compensatory increases, vitiligo melanocytes had the lowest ATP production compared to healthy melanocytes. The dysregulated mitochondria therefore lead to an imbalanced bioenergetic metabolism contributing to the pathogenesis of vitiligo [18]. The study also predicted that ROS production could be a culprit leading to this imbalance. This idea was further advocated in another study that found that skin biopsies that were taken from lesioned skin in vitiligo patients versus healthy controls had increased levels of mitochondrial ROS production in melanocytes by 257% [62]. As discussed previously, ROS production can have countless detrimental effects on mitochondrial integrity.

Another study explored the role of transient receptor potential cation channel subfamily M member 2 (TRPM2) overexpression in the pathogenesis of vitiligo [63]. TRPM2 is sensitive to oxidative stress and is found to be overexpressed in lesional melanocytes of vitiligo. The study used hydrogen peroxide to promote TRPM2 expression and found that TRPM2 mediated the calcium influx into the mitochondria, inducing apoptosis. When a specific mitochondrial Ca^2+^ uptake inhibitor was given, the melanocytes were protected from apoptosis and mitochondrial damage caused by hydrogen peroxide. Thus, the findings demonstrated that oxidative stress promotes the expression of TRPM2 and causes mitochondria-dependent apoptosis of melanocytes through calcium influx [63].

Another study looked at the expression and the activity of nicotinamide adenine dinucleotide (NAD^+^)-dependent deacetylase Sirtuin3 (SIRT3) in vitiligo melanocytes [64]. SIRT2 is responsible for regulating mitochondrial fusion via deacetylating OPA1. OPA1 affects mitochondrial respiratory chain complexes by affecting the cristae of mitochondria. SIRT3 deficiency contributed to oxidative stress-induced melanocyte apoptosis through exacerbation of mitochondrial damage and the release of cytochrome c. Thus, activation and interaction of SIRT3-OPA1 can protect vitiligo melanocytes against oxidative stress by uncoupling mitochondrial dynamics that lead to apoptotic cascades in melanocytes in vitiligo [64].

Lastly, a 2023 review article further summarized the role of mitochondrial dysfunction in the pathogenesis and development of vitiligo [19]. The article found that melanocytes from the perilesional skin of patients with vitiligo contained fewer mitochondria and were characterized by larger size and irregular structure. Furthermore, the mitochondria depicted defective TCA cycles leading to excessive accumulation of ROS and decreased ATP. Keratinocytes in perilesional vitiligo lesions also showed abnormal mitochondrial structure; specifically, they depicted rearranged cristae and increased overall mitochondrial size. Lastly, fibroblasts in perilesional skin have depicted premature senescence and increased oxidative stress [19].

#### 2.3.4. Atopic Dermatitis

Atopic dermatitis is known to be caused by epidermal barrier defects and cutaneous inflammation and physically presents as eczematous skin lesions. It is often associated with food allergies, asthma, or allergic rhinitis [65]. A recent study in 2022 also described a connection between mitochondrial abnormalities to atopic dermatitis (AD). The study performed a quantitative proteomic analysis of the epidermis from biopsies taken from patients with AD and healthy controls [65]. Key findings revealed an impairment of the NRF2-antioxidant pathway and decreased levels of mitochondrial proteins in AD patients compared to controls [65]. Both findings may contribute to the continued pro-inflammatory pathway involved in AD.

A separate study revealed an upregulation of mitochondrial activity in nonlesional AD [20]. The energy metabolism and the oxidative stress response were studied in keratinocytes from patients with nonlesional AD or healthy controls. Nonlesional AD keratinocytes showed an increase in mitochondrial oxidation of very long-chain fatty acids with enhanced complex I and II activities. An increase in aerobic metabolism resulted in oxidative stress in nonlesional AD keratinocytes, as observed by increased ROS levels and increased mitochondrial proton leak. Nonlesional AD human epidermal equivalents showed increased complex II activity and enhanced oxidative stress response. Application of MitoQ improved the AD profile in nonlesional AD human epidermal equivalents. These findings suggest that enhanced mitochondrial function fulfills metabolic requirements in nonlesional AD but leads to enhanced oxidative stress [20].

Another study found that an excessive production of hydrogen peroxide in mitochondria contributes to atopic dermatitis [66]. The study found increased levels of SOD2 and hydrogen peroxide in the mitochondria of flaky tail mouse keratinocytes. Cytochrome c levels were also markedly increased in the epidermis of the mice. After topical application of MitoQ to flaky tail mice skin, there was a reduction in inflammation and restoration of epidermal homeostasis. These findings suggest that there is an increase in SOD2 and cytochrome c in the epidermis of AD patients and a reduction in the antioxidant response as a result of mitochondrial dysfunction [66].

### 2.4. Mitochondria and Skin Cancer

Although mitochondrial involvement in skin cancer has been investigated, further research is still required as a clear understanding has not been established. One study in 2004 examined *mtDNA* mutations in light-associated skin tumors [67]. The disease processes that the study focused on included actinic keratosis, squamous cell carcinoma (SCC), and basal cell carcinoma (BCC). Actinic keratosis, although itself benign, is considered a premalignant condition that can progress to SCC over time. Phenotypically, actinic keratosis lesions present as irregular red, scaly papules or plaques that are located on sun-exposed regions of the body [68]. Invasive SCC can initially present as white, yellow keratin masses, but often progress to be ulcerated lesions and possibly patchy, papulonodular, or exophytic [69]. Finally, BCC is the most common skin cancer in humans and presents as flesh or pink-colored pearly papules. These lesions typically have central ulceration with rolled borders and telangiectasias [70]. In total, 86 facial skin samples (including actinic keratosis, squamous cell carcinoma (SCC), basal cell carcinoma (BCC), sun-exposed normal skin, and non-sun-exposed skin) were collected from 77 patients [67]. The investigators applied a polymerase chain reaction technique to determine the frequency of *mtDNA* length mutations in each sample. Results indicated that there were no significant differences in 4977 bp and 7436 bp deletions and tandem duplications in AK, BCC, SCC (light-associated skin tumors) skin samples compared to sun-exposed normal skin (*p* > 0.05) [67]. However, light-associated skin tumors did have significantly more 4977 bp deletions, 7436 bp deletions, and tandem duplications when compared to non-sun-exposed skin [67]. Overall, this suggests that sun exposure may lead to increased *mtDNA* length mutations; however, the contribution to the development of skin cancers is not clear and requires further research. In the future, studies should compare skin cancers and light-exposed normal skin against non-light-exposed normal skin.

Another study also explored *mtDNA* mutation involvement in head and neck squamous cell carcinomas. The researchers obtained 67 primary head and neck SCC tissue samples from 56 patients [71]. The aim was to detect any difference in *mtDNA* and nuclear microsatellite instability (nMSI) via PCR techniques. The authors found that 42% of the primary tumor samples contained a mitochondrial microsatellite instability in two parts of the mitochondrial D loop, 36% of the samples contained a low nMSI, and 13% contained a high nMSI [71]. Additionally, a de novo Δ4977 *mtDNA* deletion was located in 25% of the SCC samples [71]. This suggests a clear involvement of mitochondrial abnormalities in SCC. Another study in 2016 further supported the Δ4977 *mtDNA* deletion finding. The authors found significantly higher levels of the mutation in leucocytes analyzed from serum samples from 206 melanoma patients compared to 219 healthy controls [72]. Researchers concluded that having a Δ4977 *mtDNA* deletion increases the risk for melanoma [72].

### 2.5. Hair Abnormalities

Age-related hair loss is similarly mediated by mitochondrial function [73]. The anagen phase of hair growth requires rapid cell division in the hair bulb and dermal papilla [74], increasing the energy requirement and reliance on mitochondrial respiration. Further depicting the integral role of mitochondrial function and the hair cycle, a 2016 study found hair follicle stem cell differentiation activates mitochondrial aerobic respiration; conversely, mitochondrial dysfunction impairs hair regeneration [75].

The authors analyzed telogen bulge cells and anagen matrix cells to compare mitochondrial morphology and activity [75]. Pyruvate dehydrogenase kinase (PDK) and pyruvate dehydrogenase (PDH) levels were measured to estimate energy metabolism during differentiation. Increased PDK was observed in hair follicle stem cells, whereas increased PDH was observed in differentiated cells, signifying a switch from glycolysis to oxidative phosphorylation upon stem cell differentiation. Furthermore, the authors found that in vivo administration of a mitochondrial respiration inhibitor in differentiated hair follicle cells repressed hair regeneration [75]. These results demonstrate the integral role of mitochondrial respiration in hair follicle stem cell differentiation and hair regeneration.

In addition to parameters of skin aging, the study conducted by Singh et al. also assessed the effect of mitochondrial dysfunction on hair loss [36]. In this study, *mtDNA* depletion was induced in some mice, leading to a reduction in their *mtDNA* levels. The authors defined *mtDNA*-depleted mice as mice with reduced *mtDNA* content and gene expression. These mice depicted dysfunctional hair follicles and visible hair loss. Reduced hair density, hair loss, and hair graying were observed four weeks after turning on mutant transgene expression. Overall, 100% of *mtDNA*-depleted mice depicted the hair loss phenotype compared to 0% of wild-type mice. Interestingly, male mice showed dispersed hair loss, whereas female mice demonstrated time-dependent hair loss patterns of increased severity compared to males. The authors postulate that hair loss patterns may differ according to biological sex due to the regulation of mitochondrial functions by sex hormones [36]. However, hair pathology was reversed upon turning off mutant transgene expression, thereby restoring mitochondrial function. Thus, this study demonstrated the reversibility of hair loss upon restoration of mitochondrial function and highlighted the potential utility of mitochondria-targeted therapeutic strategies for hair regeneration.

The 2020 study conducted by Wu et al. assessed the utility of allogeneic mitochondrial transplantation in the restoration of hair growth, in addition to parameters associated with cutaneous aging, as previously described [73]. Following removal of dorsal hair, 100-week-old mice subsequently received weekly unilateral injections of either allogeneic mitochondria-labeled 5-bromo-2′-deoxyuridine, with or without Pep-1 conjugation (P-Mito and Mito groups, respectively), or human platelet-rich plasma. Contralateral sides were left untreated. Although all treatments simulated hair regrowth, the authors found that only P-Mito maintained hair length until day 28, depicting the efficacy of mitochondrial transplantation in hair regrowth [73]. Collectively, these studies depicted the integral role of mitochondrial respiration in proper hair growth and highlighted the potential utility of mitochondrial transplantation in restoring hair growth. However, further studies must be conducted to determine whether mitochondrial transplantation can mitigate hair loss in humans, as demonstrated with mouse models. Study results for each evaluated cutaneous condition are summarized in Table 1.

## 3. Discussion

As mitochondria are important energy-producing organelles, mitochondrial dysfunction can greatly impact labile cells, including cutaneous epithelial cells. This review of the literature depicts the importance of mitochondrial integrity for the regulation of cutaneous aging, photoaging, wound healing, skin cancer, hair abnormalities, and various specific dermatologic disorders with mitochondrial involvement, as mitochondrial dysfunction and mutation accumulation have been associated with changes in each of these dermatologic phenomena. Furthermore, mouse model studies have better established a link between mitochondrial dysfunction and various conditions, demonstrating the direct effects of *mtDNA*-depleted mice or mutant knock-in mice. For example, *mtDNA* mouse models depicted signs of premature aging [21,22,42], skin wrinkling [36], and hair loss [36]. Signs of premature aging included weight loss, reduced subcutaneous fat, alopecia, kyphosis, osteoporosis, anemia, reduced fertility, and heart enlargement [22], while signs of premature cutaneous aging included increased collagen-degrading metalloproteases and a downregulation of collagen biosynthesis genes, which may contribute to wrinkle formation [42]. Remarkably, Singh et al. also demonstrated phenotypic reversibility of cutaneous aging and hair loss upon restoration of mitochondrial function [36].

Additionally, aberrant mitochondrial involvement in dermatological disorders has been well established. Several common conditions including SLE, AD, psoriasis, and vitiligo have each shown to have some association with mitochondrial dysfunction. For instance, one study found defective mitochondrial ATP production with reciprocal increases in glycolytic activity in cultured epidermal vitiligo melanocytes [18]. Abnormal ATP production was also found to occur in SLE [52]. Studies have also shown that *mtDNA* mutations also have a role in the development of skin cancers including SCC, BCC, AK, and melanoma; however, a more clear relationship must be established [67,71,72].

A greater understanding of the causative role of mitochondria in various dermatologic phenomena can inspire potential therapeutic strategies. Studies have assessed the effects of topical CoQ_10_ application [25] and oral CoQ_10_ supplementation [76] on mitochondrial function and various skin parameters implicated in aging. Topical CoQ_10_ supplementation resulted in a significant 44% improvement of mitochondrial membrane potential compared to untreated controls [25], and oral supplementation reduced seasonal deterioration of viscoelasticity, wrinkles, and microrelief lines, and improved skin smoothness [76]. Furthermore, a 2020 mouse model study found allogeneic mitochondrial transplantation to reduce the expression of gene markers associated with aging, suggesting artificial or allogeneic mitochondria transfer may be a potential therapeutic strategy warranting further research.

This review highlights the vital role of mitochondria in dermatologic conditions, marking mitochondria as a promising target for therapeutic strategies. Additional studies are required to further assess the effect of mitochondrial dysfunction. However, preliminary studies and mouse model studies depict promising results of various mitochondrial interventions on cutaneous aging, photoaging, hair loss, and wound healing. Ultimately, future clinical studies will allow for better understanding of how therapies directed at mitochondrial function may have clinical benefit in dermatological conditions.

## 4. Conclusions

As energy-rich cells, cutaneous epithelial and hair follicle dermal papilla cells may be readily impacted by mitochondrial dysfunction or mutation. Studies have found associations of mitochondrial damage or altered activity with phenotypic signs of cutaneous aging, hair loss, impaired wound healing, systemic lupus erythematosus, psoriasis, vitiligo, and atopic dermatitis. Murine studies have established causality and reversibility for some conditions, such as hair loss. As such, components of mitochondria appear a promising therapeutic target for the management of dermatologic disease.

## Figures and Tables

**Table 1:** Summary of Study Findings for Each Evaluated Cutaneous Condition.

Cutaneous Parameter	Summary of Literature Review
Chronological aging	- Age-related increase in the 4977 bp *mtDNA* deletion in normal skin - Significantly decreased mitochondrial membrane potential and increased ROS levels among older vs. younger fibroblast donors - Age-related decrease in fibroblast complex II activity - Age-related impairment in Coenzyme Q10 observed in various organs
Photoaging	- Greater accumulation of *mtDNA* deletions in sun-exposed skin vs. protected skin, including 10-fold increase in the common deletion - Earlier UV radiation was associated with a greater incidence of UVA-induced *mtDNA* deletions, regardless of whether UVA exposure was sustained - mtDNA-depleted skin fibroblasts depicted increased levels of collagen-degrading metalloproteases and downregulated collagen biosynthesis genes
Wound healing	- Wound stage-specific changes in mitochondrial metabolism - Early-stage wound macrophages characterized by mitochondrial ROS production and HIF1a stabilization - Late-phase resolving macrophages marked by IL-4Ra-mediated mitochondrial respiration - Mitochondrial dysfunction is associated with delayed wound healing in diabetic mice
Systemic Lupus Erythematosus	- SLE T cells contain mitochondria with excessive ROS production, low glutathione and ATP levels, and hyperpolarized transmembrane potentials, increasing susceptibility of T-cell death - An accumulation of oxidized nucleoids in SLE triggers a type I interferon response upon extrusion - Programmed mitochondrial removal typical in erythropoiesis is defective in SLE, resulting in an accumulation of RBCs carrying mitochondria which may be engulfed by macrophages - Lupus low-density granulocytes had increased mitochondrial ROS, promoting *mtDNA* oxidation and neutrophil extracellular trap formation
Psoriasis	- Significantly decreased gene expression of uncoupling protein 2, dynamin-related protein 1, and calcineurin (all regulators of mitochondrial function) in those with psoriasis vs. controls - Imiquimod-induced psoriasiform skin inflammation resulted in increased production of mitochondrial ROS and inflammatory cytokines
Vitiligo	- Abnormalities in mitochondrial ATP production with a compensatory increase in glycolytic enzymes observed in epidermal vitiligo melanocytes vs. healthy melanocytes - Vitiligo melanocytes had lower ATP production compared to healthy melanocytes - Vitiligo melanocytes had greater proton leak, suggesting an impaired ETC function - Activation and interaction of SIRT3-OPA1 protected vitiligo melanocytes against oxidative stress by uncoupling mitochondrial dynamics that led to apoptotic cascades in vitiligo melanocytes - Melanocytes from perilesional skin of patients with vitiligo contained fewer, larger, and irregularly structured mitochondria - Perilesional mitochondria depicted defective TCA cycles leading to excessive accumulation of ROS and decreased ATP - Perilesional keratinocytes showed abnormal mitochondrial structure with rearranged cristae and increased overall mitochondrial size
Atopic dermatitis	- Atopic dermatitis patients depicted impairment of the NRF2-antioxidant pathway and decreased levels of mitochondrial protein compared to controls - Lesional epidermis was associated with an increase in SOD2 and cytochrome c and a reduction in antioxidant response as a result of mitochondrial dysfunction in flaky tail mouse keratinocytes
Skin cancer	- One study found no significant differences in 5977 bp and 7436 bp deletions and tandem duplication in skin samples from AK, BCC, SCC vs. normal skin - Another study found significantly higher levels of the 4977 *mtDNA* mutation in leucocytes analyzed from serum samples of 206 melanoma patients vs. 219 healthy controls
Hair	- Mitochondrial dysfunction impaired hair regeneration - There was a switch from glycolysis to oxidative phosphorylation upon stem cell differentiation - In vivo administration of a mitochondrial respiration inhibitor in differentiated hair follicle cells repressed hair regeneration - *mtDNA* depletion was induced in some mice, which depicted dysfunctional hair follicles and visible hair loss. Hair pathology was reversed upon turning off mutant transgene expression

## Data Availability

No new data were created or analyzed in this study. Data sharing is not applicable to this article.

## Abbreviations

AD	Atopic Dermatitis
ATP	Adenosine triphosphate
BCC	Basal cell carcinoma
ETC	Electron transport chain
*mtDNA*	Mitochondrial DNA
PDH	Pyruvate dehydrogenase
PDK	Pyruvate dehydrogenase kinase
ROI	Reactive oxygen intermediates
ROS	Reactive oxygen species
SCC	Squamous cell carcinoma
SLE	Systemic lupus erythematosus
